# Nephropathy in Hypertensive Animals Is Linked to M2 Macrophages and Increased Expression of the YM1/Chi3l3 Protein

**DOI:** 10.1155/2019/9086758

**Published:** 2019-07-10

**Authors:** Paula Andréa Malveira Cavalcante, Natalia Alenina, Alexandre Budu, Leandro Ceotto Freitas-Lima, Thaís Alves-Silva, Juan Sebastian Henao Agudelo, Fatimunnisa Qadri, Niels Olsen Saraiva Camara, Michael Bader, Ronaldo Carvalho Araújo

**Affiliations:** ^1^Department of Biophysics, Federal University of São Paulo (UNIFESP), São Paulo, SP, Brazil; ^2^Max-Delbrück-Center for Molecular Medicine (MDC), Berlin, Germany; ^3^Laboratory of Exercise Genetics and Metabolism, Federal University of São Paulo (UNIFESP), São Paulo, SP, Brazil; ^4^German Center for Cardiovascular Research (DZHK), Partner Site Berlin, Germany; ^5^Department of Immunology, Institute of Biomedical Sciences, University of São Paulo, São Paulo, Brazil; ^6^Laboratory of Renal Pathophysiology, Department of Medicine, School of Medicine, University of São Paulo, São Paulo, Brazil; ^7^Institute for Biology, University of Lübeck, Germany; ^8^Berlin Institute of Health (BIH), Berlin, Germany; ^9^Charité University Medicine, Berlin, Germany

## Abstract

Macrophages contribute to a continuous increase in blood pressure and kidney damage in hypertension, but their polarization status and the underlying mechanisms have not been clarified. This study revealed an important role for M2 macrophages and the YM1/Chi3l3 protein in hypertensive nephropathy in a mouse model of hypertension. Bone marrow cells were isolated from the femurs and tibia of male FVB/N (control) and transgenic hypertensive animals that overexpressed the rat form of angiotensinogen (TGM(rAOGEN)123, TGM123-FVB/N). The cells were treated with murine M-CSF and subsequently with LPS+IFN-*γ* to promote their polarization into M1 macrophages and IL-4+IL-13 to trigger the M2 phenotype. We examined the kidneys of TGM123-FVB/N animals to assess macrophage polarization and end-organ damage. mRNA expression was evaluated using real-time PCR, and protein levels were assessed through ELISA, CBA, Western blot, and immunofluorescence. Histology confirmed high levels of renal collagen. Cells stimulated with LPS+IFN-*γ* in vitro showed no significant difference in the expression of CD86, an M1 marker, compared to cells from the controls or the hypertensive mice. When stimulated with IL-4+IL-13, however, macrophages of the hypertensive group showed a significant increase in CD206 expression, an M2 marker. The M2/M1 ratio reached 288%. Our results indicate that when stimulated *in vitro*, macrophages from hypertensive mice are predisposed toward polarization to an M2 phenotype. These data support results from the kidneys where we found an increased infiltration of macrophages predominantly polarized to M2 associated with high levels of YM1/Chi3l3 (91,89%), suggesting that YM1/Chi3l3 may be a biomarker of hypertensive nephropathy.

## 1. Introduction

Recent studies have established a strong association between immunoinflammatory processes, hypertension, and chronic forms of kidney disease [1–4]. These pathologies are marked by progressive renal fibrosis and ultimately organ failure [5, 6]. Nephropathy in the wake of sustained hypertension is the second leading cause of end-stage renal disease (ESRD), a condition whose incidence is increasing worldwide [7, 8]. Studies of both animal models of CKD and human hypertension have revealed high levels of proinflammatory cytokines and have exposed inflammation as the most significant factor in the progression of fibrosis, regardless of the initial cause [9, 10]. Thus, immunoinflammatory mechanisms are now recognized as crucial contributors to both acute and chronic forms of kidney disease [3].

Hypertension is marked by an infiltration of immune cells into the kidneys, vessel walls, perivascular regions, and nervous system; simultaneously, there is a high release of cytokines, a production of reactive oxygen species (ROS), and an increase in the expression of adhesion molecules [4, 11, 12]. These events are mediated by the innate and adaptive immune systems and have been shown to contribute to the sustained elevation of blood pressure [4].

Several studies [9, 10, 11] have implicated macrophages in the pathogenesis of hypertension. An elegant study by Wenzel et al. [13] provided strong evidence that monocytes and macrophages mediate angiotensin II- (Ang II-) induced hypertension and vascular dysfunction.

Studies of hypertension have revealed that Ang II activates angiotensin II receptor type 1 (AT1R). Upon hemodynamic injury, this leads to the recruitment of monocytes to the vasculature, kidney, and heart [13–16]. After infiltration, monocytes differentiate into at least two phenotypes: M1 or M2 [17, 18].

M1 cells express high levels of proinflammatory cytokines including interleukin-1 beta (IL-1*β*) and tumor necrosis factor-alpha (TNF*α*) and produce high amounts of ROS, which strongly promote microbicidal and tumoricidal activity. In contrast, M2 macrophages, also known as “alternatively activated,” have anti-inflammatory effects and mediate tissue repair through the secretion of IL-10 and transforming growth factor-beta (TGF-*β*) [19, 20].

The M2 response has been shown to depend on a sustained stimulus. Persistent lesions also cause irreversible fibrosis and the destruction of tissue [21, 22]. Studies have shown that the M2 phenotype enters a prorepair stage with the expression of chitinase-like protein-3 (YM1/Chi3l3) and acquires pathogenic functions [23–26].

YM1/Chi3l3 is a marker expressed by M2 macrophages in diverse tissues in the mouse and has been associated with recovery and function restoration [27, 28]. This protein displays chemotactic activity for T lymphocytes, bone marrow cells, and eosinophils [29]. Here, we attempt to determine whether the YM1/Chi3l3 marker has functions in arterial hypertension and hypertensive nephropathy, where its roles have not yet been clarified.

YM1/Chi3l3 exhibits a significant homology to microbial chitinases and several “chitinase-like” proteins reported recently (in tissues including human cartilage- (HC-) gp39, human macrophage chitotriosidase, porcine smooth muscle gp38k, and Drosophila DS-47) [30]. A recent study [31] has shown that YKL-40 serve as a new biomarker for predicting hypertension in a population of prehypertensive subjects.

As Ang II promotes macrophage recruitment [32, 33], we decided to study the cells in a mouse model in which the renin-angiotensin system could be controlled. Ang II arises from the precursor angiotensinogen (AOGEN), making it one of the most important factors in the regulation of human blood pressure.

Mice lacking AOGEN presented drastic hypotension, pathomorphological alterations in the kidney, and reduced survival [34–36]. In contrast, transgenic mice overexpressing the rat AOGEN gene (TGM(rAOGEN)123, TGM123-FVB/N) developed hypertension, cardiac hypertrophy, impaired heart function, high levels of albuminuria, and pronounced fibrosis [34, 37, 38]. This suggests that besides being a good model of arterial hypertension, this animal model can also be used in studies of hypertensive nephropathy. We hypothesized that macrophages of these hypertensive animals from 10 to 12 weeks of age would be predisposed to polarize to an M2 phenotype and high levels of YM1/Chi3l3 would be found in their kidneys. This could make the protein a marker for hypertensive nephropathy.

## 2. Materials and Methods

### 2.1. Animals

The study was approved by the Federal University of São Paulo Ethics Committee (approval number CEUA 2384220216 in 29/Feb/2016). 10- to 12-week-old hypertensive (TGM123-FVB/N) male mice and normotensive controls (FVB/N) were used in the experiments. Mice overexpressing rat AOGEN (TGM(rAOGEN)123), originally generated on NMRI background [37], were crossed with FVB/N mice for 8 generations to transfer the rAOGEN transgene to the FVB/N background and generate the hypertensive model. The animals were maintained under standardized conditions with an artificial 12 h dark-light cycle and free access to food and water. Mice from the control group (FVB/N) (*n* = 9) and hypertensive group (TGM123-FVB/N) (*n* = 9) were euthanized by cervical dislocation; then, the femurs, tibia, and kidneys were extracted.

The transgenic animals used in this study (TGM123-FVB/N) are considered a valid model of arterial hypertension and hypertensive nephropathy since they presented mean blood pressure around 158 mmHg in males and 132 mmHg in females and developed high levels of albuminuria and pronounced renal fibrosis [34, 37, 38].

### 2.2. Cell Culture

Bone marrow-derived macrophages (BMDM) were isolated from the femur and tibia of the control (FVB/N) and hypertensive (TGM123-FVBN) male mice. Cells were filtered using a Cell Steiner 70 *μ*m filter (Corning, USA), and the flow-through cells were washed twice with PBS by centrifugation at 300 g for 5 min. Subsequently, cells were lysed with 0.83% NH_4_Cl (3 min/4°C) and cultured in RPMI 1640 (Gibco) and DMEM High Glucose Medium (Gibco) supplemented with 10% FBS (Gibco), 1% penicillin-streptomycin (Gibco), and murine M-CSF (macrophage colony-stimulating factor) (PeproTech, USA). The culture medium was refreshed on day 3 and maintained until day 7 to promote BMDM differentiation. On day 8, the cells were polarized to M1 by 10 *μ*g/ml IFN-*γ* (R&D Systems) and 1 mg/ml LPS (*E. coli*-LPS, Sigma-Aldrich, USA) and to M2 by 10 *μ*g/ml IL-4 (R&D Systems) and 10 *μ*g/ml IL-13 (R&D Systems). After induction of polarization, cells were cultured for 48 h and thereafter used for the RNA extraction, one well per condition per animal.

### 2.3. Real-Time Quantitative PCR

Real-time quantitative PCR (qPCR) was used to evaluate the mRNA expression of macrophage polarization marker genes. RNA isolation from the kidney and the macrophage cultures was performed using TRIzol (TRIzol Reagent, Invitrogen, Germany) according to the manufacturer's instructions. The RNA pellet was resuspended in RNase-free water and kept at −80°C until used. RNA concentration was quantified using spectrophotometry (NanoDrop, München, Germany), and 1 *μ*g of RNA was taken for the synthesis of cDNA using M-MLV Reverse Transcriptase (Invitrogen). The reaction product was amplified using the GoTaq qPCR Master Mix (Promega; Germany) by real-time quantitative PCR (ABI 7900HT Real-Time PCR System, Applied Biosystems, Germany). The gene-specific primer sequences are listed in Table 1.

### 2.4. Histology

Paraffinized sections of renal tissue (5 *μ*m thick) were deparaffinized, rehydrated, and incubated with 1% sirius red in a saturated solution of picric acid for 60 min. Unbound sirius red was removed by treating the sections with acidified water and coverslipped using Eukit. The sections were examined and photographed at a magnification of 2x with a Keyence BZ-9000 microscope (Keyence, Germany). The quantification of fibrosis was performed using the Keyence digital image analysis software (Keyence BZII).

### 2.5. Protein Extraction

Kidney tissue (ca. 1 g) was homogenized in extraction buffer containing phosphatases and protease inhibitors 100 mM Tris-HCl (pH 7.5), 1% Triton X-100, 10% sodium dodecyl sulfate (SDS), 10 mM EDTA, 100 mM sodium fluoride, 10 mM sodium pyrophosphate, 10 mM sodium orthovanadate, 2 mM phenylmethylsulfonyl fluoride (PMSF), and 0.1 mg aprotinin/ml at 14000 rpm by 40 minutes at 4°C. Protein concentrations were determined by the Bradford assay (Bio-Rad Laboratories, Hercules, CA, USA). Extracts were used for Western blotting, CBA, and ELISA analysis.

### 2.6. Enzyme-Linked Immunosorbent Assay (ELISA)

Renal levels of IL-10, IL-1*β*, and TNF*α* were determined using Quantikine Mouse ELISA kits (R&D Systems, MN, USA) according to the manufacturer's instructions.

### 2.7. Detection of TGF-*β* and YM1/YM2 by Western Blot (WB)

Proteins extracted from the mouse kidney were submitted to SDS-PAGE (25 *μ*g of protein/well) and transferred to a PVDF membrane at 300 mA, for 2 h, in ice-cold buffer (3 g/l Tris, 14,4 g/l glycine, and 20% methanol). Membrane blocking was executed overnight, at 4°C, in PBS-T (137 mM NaCl, 2.7 mM KCl, 8.1 mM Na_2_HPO_4_, 1.5 mM KH_2_PO_4_, 0.05% Tween-20, pH 7.2), containing 5% (*m*/*v*) of bovine serum albumin (Sigma). The membrane was then incubated with 1 : 1000 anti-TGF-*β* antibody (Cell Signaling Technology, #3709) in PBS-T or anti-YM-1+YM-2 and 1 : 10000 (Abcam, #ab192029) in PBS-T+5% BSA overnight at room temperature on a shaker. After washing the membrane (three times, 10 min), the secondary antibody (anti-IgG rabbit, HRP-conjugated, Sigma, #A6154, 1 : 5000) was added and incubation proceeded for 1 h on a shaker. After three washes as above, the substrate SuperSignal West Pico (Pierce) was used to detect the bands in an imager. The antibodies were removed from the membrane with two subsequent 10-minute incubations in mild stripping buffer (15 g/l glycine, 1 g/l SDS, 10 ml Tween-20, pH 2.2). The membrane was washed twice (10 min each) with PBS and twice (5 min each) in PBS-T, blocked as above, and incubated for 2 h in PBS-T with the primary anti-*β*-actin antibody (1 : 5000, raised in rabbit) used as a loading control. Secondary antibody incubation and detection of the bands were performed as above. The densitometry was obtained using the software Scion Image (Release Alpha 4.0.3.2), and relative protein expression was determined by dividing TGF-*β* and YM1/YM2 by *β*-actin densitometry data.

### 2.8. Cytokine Assessment in Kidney Sample (CBA)

Levels of concentrations of interleukin-6 (IL-6), Monocyte Chemoattractant Protein-1 (MCP-1), Interferon-*γ* (IFN-*γ*), and tumor necrosis factor (TNF) in a kidney sample were measured using the BD™ CBA Mouse Inflammation Kit (Becton Dickinson (BD), USA). Controls and samples were processed according to the manufacturer's instructions. The results were normalized according to the total value of the protein and expressed as pg/mg protein.

### 2.9. Immunofluorescence (IF)

Immunofluorescence for F4/80, iNOS, and YM1 was performed by incubating the sections with Alexa Fluor 594 (1 : 300, ThermoFisher, #A11007) anti-mouse and Alexa Fluor 488 anti-rabbit (1 : 300, ThermoFisher, #A11034) anti-mouse sections. The nuclei were stained with DAPI (1 : 600, ThermoFisher, #D1306). The kidney slices were incubated with primary mouse anti-F4/80 antibodies (1 : 500, Abcam, #ab6640) overnight at 4°C, rabbit anti-iNOS (1 : 100, Abcam, #ab15323), and rabbit anti-YM-1+YM-2, (1 : 10000, Abcam, #ab192029). Nonspecific binding was controlled by the replacement of a negative control by the primary antibody.

### 2.10. 3D Confocal Microscopy

Immunopositive signals were detected by 3D confocal microscopy (Zeiss LSM 780, Germany). The images were analyzed with ImageJ software.

The images were acquired employing a PlanNeofluar 40x objective with 1.3 numerical aperture. DAPI, Alexa 594, and Alexa 488 were excited with 405 nm, 594 nm, and 488 nm lasers, while emission was collected between 421 nm-488 nm, 597 nm, and 646 nm and 498 nm and 554 nm, respectively. Slices on the *Z* plane were taken. The stacked images were rendered at the maximum precision available, and three-dimensional projection was performed using the “surface” option (ZEN software, Carl Zeiss, Germany).

### 2.11. Statistical Analysis

Data are presented as means ± SEM. The statistical analysis was performed with Prism software (GraphPad Software, La Jolla, CA, USA). Multiple groups were compared through a one-way analysis of variance (ANOVA) followed by the Bonferroni post hoc test. The two-group analysis was performed using Student's *t*-test. *p* values < 0.05 were considered statistically significant.

## 3. Results

### 3.1. The Macrophages of Hypertensive Animals Have a Predisposition toward the M2 Phenotype

**Figure 1:**
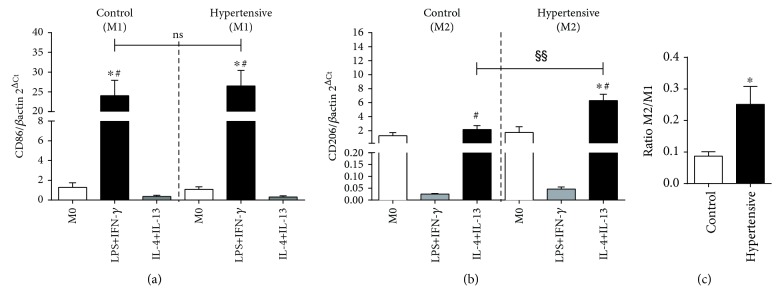
The macrophages of hypertensive animals have a predisposition toward the M2 phenotype. Four mice for each group, 6 wells per animal. (a) CD86 gene expression. Values were expressed as the mean ± SEM. ^∗^*p* < 0.05 compared to M0 groups and ^#^*p* < 0.05 relative to IL-4+IL-13 groups, with ANOVA followed by Bonferroni correction for multiple comparisons. (b) CD206 gene expression. Values expressed as the mean ± SEM. ^∗^*p* < 0.05 compared to M0 groups, ^#^*p* < 0.05 relative to LPS+IFN-*γ* groups, and ^§§^*p* < 0.05 compared to IL-4+IL-13 from control mice, with ANOVA followed by Bonferroni correction for multiple comparisons. (c) Ratio M2/M1. Values are expressed as the mean ± SEM. ^∗^*p* < 0.05 compared to the control group with the *t*-test. The M2/M1 ratio reached 288%.

Initially, we cultured macrophages extracted from the bone marrow of hypertensive TGM123-FVB/N and control mice (FVB/N) over 10 days. After *in vitro* stimulation, we observed no differences in the expression of CD86 (an M1 marker) between the groups (Figure 1(a)). The hypertensive group, however, revealed a significant increase in the expression of CD206 (M2 marker) compared to controls (Figure 1(b)). In this group, the M2/M1 ratio reached 288% (Figure 1(c)). These results showed that the macrophages of hypertensive animals had a predisposition toward the M2 phenotype.

### 3.2. The Kidneys of Hypertensive Animals Show High Levels of Collagen and Macrophage Polarization to the M2 Phenotype

**Figure 2:**
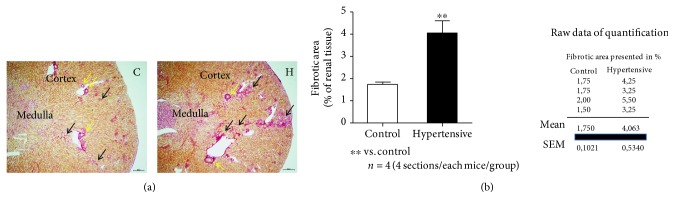
High levels of collagen were found in the kidneys of the hypertensive group. (a) Picro sirius red stained renal paraffin sections (renal cortex and medulla) of nonhypertensive control (C) and of rAOGEN transgenic hypertensive (H) mice at the age of 12 weeks. Light microscopic images were taken using a Keyence microscope (BZ-9000). Yellow arrows represent the perivascular fibrosis and black arrows the interstitial fibrosis. The scale bar = 300 *μ*m. (b) Analysis and quantification of total fibrosis (interstitial and perivascular) were performed on digital renal images using a Keyence BZII analyzer. Data were presented as the area of fibrosis in % of whole renal section (4 sections/animal/groups). Histology with four mice of each group.

Histological examinations confirmed that hypertensive animals TGM123-FVB/N exhibited higher levels of collagen, indicative of both interstitial and perivascular fibrosis, compared to control animals (FVB/N) (Figures 2(a) and 2(b)).

**Figure 3:**
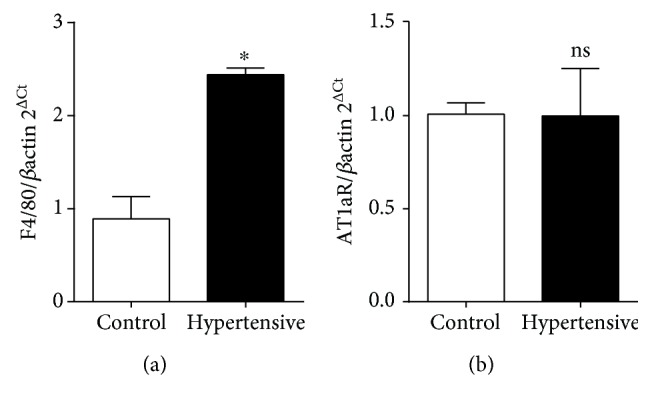
F4/80 gene expression indicates the presence of macrophages in the kidneys of hypertensive mice. Ang II levels are elevated, but Ang II is probably not binding to AT1aR. (a) F4/80 gene presented a significant difference when compared to the respective controls. Values are expressed as the mean ± SEM. ^∗^*p* < 0.05 compared to the control group with the *t*-test. (b) AT1aR gene presented no significant difference when compared to the respective controls. Values are expressed as the mean ± SEM. ^∗^*p* < 0.05 compared to the control group with the *t*-test. Five mice for each group.

We analyzed the association between F4/80 gene expression and the presence of macrophages in the kidneys of hypertensive and control mice, which revealed significant differences in the hypertensive group (Figure 3(a)). Hypertensive groups exhibited no increase in the expression of AT1aR (Figure 3(b)), suggesting that the Ang II-AT1R interaction tends to shift the M1/M2 balance toward M2 predominance.

**Figure 4:**
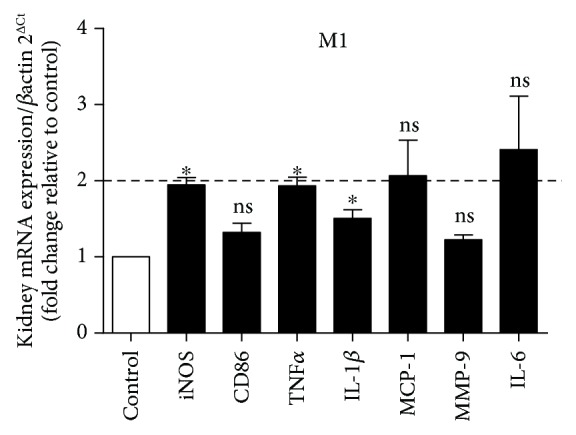
Three markers of the M1 phenotype showed an increase, but the changes were smaller than twofold. Values are expressed as the mean ± SEM. ^∗^*p* < 0.05 compared to the control group with the *t*-test. The genes iNOS, TNF*α*, and IL-1*β* presented a significant difference when compared to the respective controls. Five mice for each group.

We evaluated the expression of genes related to the polarization to M1 (iNOS, CD86, TNF*α*, IL-1*β*, MCP-1, MMP-9, and IL-6). Statistically significant differences were found for transcripts of iNOS, TNF*α*, and IL-1*β* (Figure 4). However, these differences were smaller than twofold. Karlen et al. [39] showed that real-time qPCR yields reliable estimates only in cases when the relative expression is twofold or higher.

**Figure 5:**
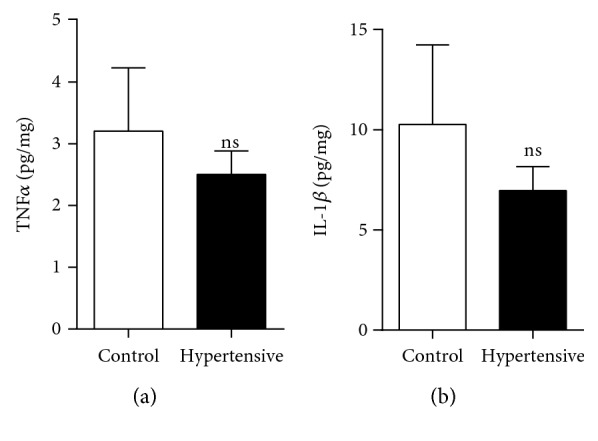
There was no increase in levels of the M1 marker proteins TNF*α* and IL-1*β*. (a) Renal levels of TNF*α* by ELISA. (b) Renal levels of IL-1*β* by ELISA. Values are expressed as the mean ± SEM. ^∗^*p* < 0.05 compared to the control group with the *t*-test. Five mice for each group.

**Figure 6:**
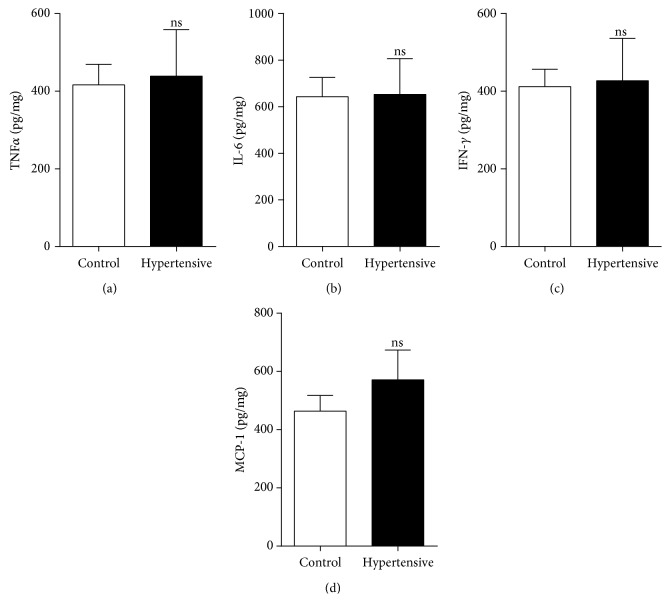
There was no increase in levels of M1 marker proteins TNF*α*, IL-6, IFN-*γ*, and MCP-1: (a) renal levels of TNF*α* by CBA; (b) renal levels of IL-6 by CBA; (c) renal levels of IFN-*γ* by CBA; (d) renal levels of MCP-1 by CBA. Values were expressed as the mean ± SEM. ^∗^*p* < 0.05 compared to the control group with the *t*-test. Five mice for each group.

**Figure 7:**
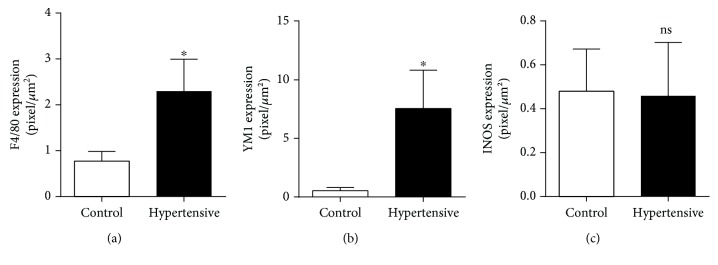
High levels of F4/80 in the hypertensive group indicate the presence of macrophages, and the YM1 marker shows the predominance for the M2 phenotype. There was no increase in iNOS, an M1 phenotype marker: (a) renal levels of F4/80 by IF; (b) renal levels of YM1 by IF; (c) renal levels of iNOS by IF. Values were expressed as the mean ± SEM. ^∗^*p* < 0.05 compared to the control group with the *t*-test. Seven sections/animal/groups. Four mice for each group.

In addition, these gene expression data do not support the results from measurements of protein levels, in which we did not find a significant difference in the M1 marker protein levels relative to the respective control groups. This was the case for TNF*α* (Figures 5(a) and 6(a)), IL-1*β* (Figure 5(b)), IL-6 (Figure 6(b)), IFN-*γ* (Figure 6(c)), MCP-1 (Figure 6(d)), and iNOS (Figure 7(c)).

**Figure 8:**
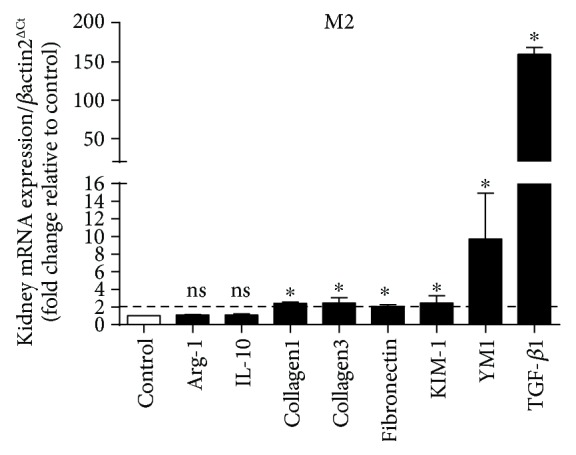
Increase in markers related to the M2 phenotype in the kidneys. The genes YM1, TGF-*β*1, KIM-1, fibronectin, type I collagen, and type III collagen presented a significant difference when compared to the respective controls, and these results were higher than twofold. Values were expressed as the mean ± SEM. ^∗^*p* < 0.05 compared to the control group with the *t*-test. Five mice for each group.

Next, we evaluated the expression of genes related to polarization to the M2 phenotype (Arg-1, IL-10, type I collagen, type III collagen, fibronectin, KIM-1, YM1, and TGF-*β*1). Statistically significant differences were found for the expression of YM1, TGF-*β*1, type I collagen, type III collagen, fibronectin, and KIM-1 (Figure 8).

**Figure 9:**
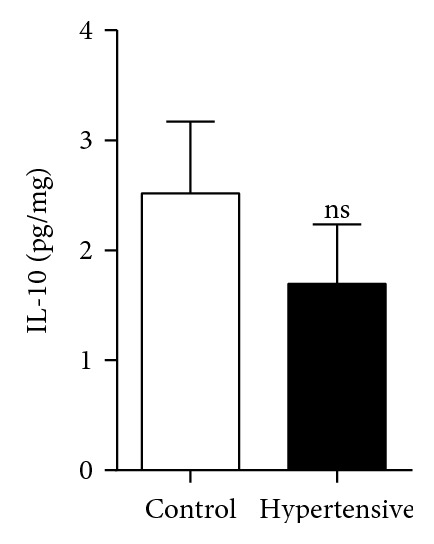
IL-10 do not represent a form of protection against renal fibrosis in the hypertensive animals. Values were expressed as the mean ± SEM. Five mice for each group.

We verified this at the protein level for IL-10, where no significant difference was again detected (Figures 8 and 9).

**Figure 10:**
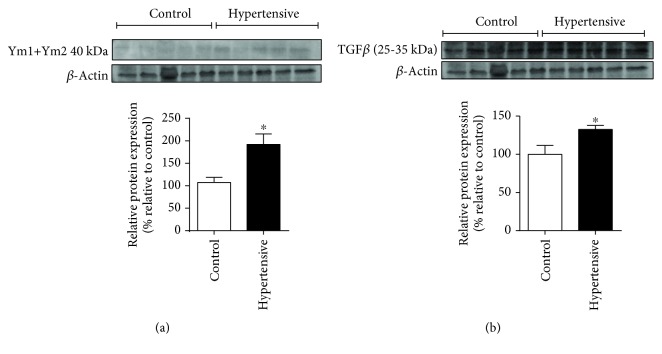
Levels of M2 marker proteins. M2 macrophages produced high levels of YM1 protein (91,89%): (a) renal levels of YM1/YM2/*β*-actin by WB; (b) renal levels of TGF-*β*1/*β*-actin by WB. Values were expressed as the mean ± SEM. ^∗^*p* < 0.05 compared to the control group with the *t*-test. Five mice for each group.

We confirmed the high levels of other M2 markers, including TGF-*β*1 and YM1, by Western blot (Figures 10(a) and 10(b)).

### 3.3. High Levels of YM1/Chi3l3 Were Found in the Kidneys of Hypertensive Animals

Alongside a general increase in F4/80 levels (Figure 7(a)), other M2 markers exhibited a sharp rise of expression in hypertensive kidneys (Figures 7, 8, 10(a) and 10(b)).

**Figure 11:**
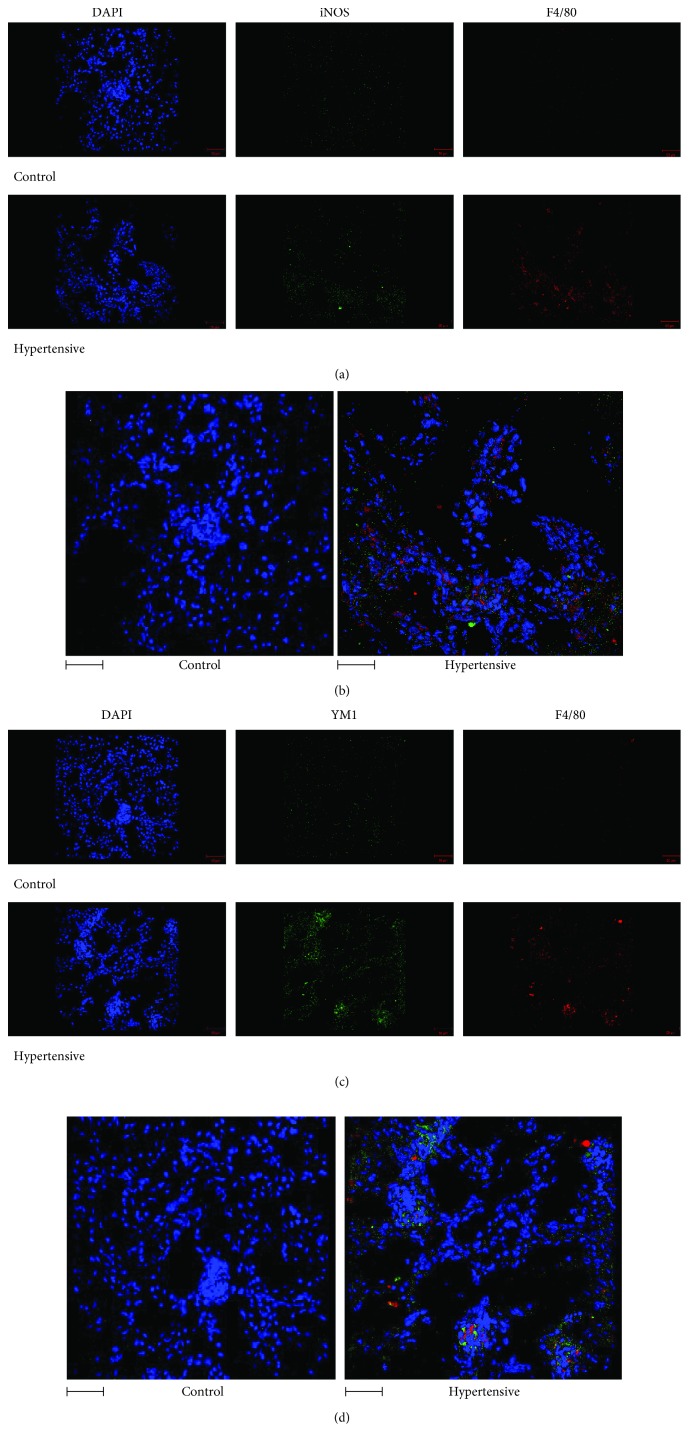
Immunofluorescence images of renal tissue showed colocalization between the F4/80, a macrophage marker, and YM1, suggesting that high levels of the YM1 protein were secreted by M2 macrophages. On the other hand, there was no increase in iNOS, an M1 marker: (a) DAPI, iNOS, and F4/80 by IF; (b) 3D overlapping images of DAPI, iNOS, and F4/80 by IF; (c) panel of DAPI, YM1, and F4/80 by IF; (d) 3D overlapping images of DAPI, YM1, and F4/80 by IF. The scale bar = 5 *μ*m.

In addition, quantitative immunofluorescence showed a significant jump in the expression of YM1 in hypertensive animals (Figure 7(b)) but no difference in the M1 marker gene iNOS (Figure 7(c)), as can be observed in Figures 11(a)–11(d).

## 4. Discussion

Despite strong evidence that macrophage polarization plays an important role in the development of hypertension [9, 10, 11], few studies have addressed the role of these cells in the disease. We carried out a study of the polarization of these cells from hypertensive animals *in vitro* and *in vivo*, with the aim of developing insights into their possible functions in the kidney and roles in renal pathologies.

This study revealed an important role for M2 macrophages during hypertensive nephropathy. Our main finding was that the kidneys of 10- to 12-week-old hypertensive TGM123-FVB/N mice exhibited high levels of collagen, indicative of perivascular and interstitial fibrosis and confirming earlier studies on this model [34, 38]. These symptoms were accompanied by an increase in the expression of marker genes for the M2 phenotype, suggesting that hypertensive kidneys had undergone an infiltration of macrophages polarized preferentially toward M2. This effect reflected the results of our *in vitro* investigation, in which macrophages from hypertensive animals also had a predisposition toward the M2 type.

Ang II, a peptide hormone whose effects are similar to those of proinflammatory cytokines, plays a key role in the progression of chronic renal damage and may be involved in the development of fibrosis [40–42]. This vasoactive peptide activates mesangial and tubular cells and interstitial fibroblasts, increasing the expression and synthesis of extracellular matrix proteins. Studies have shown that blocking Ang II action through ACE inhibitors and Ang II receptor antagonists prevents proteinuria and fibrosis, as well as the infiltration of inflammatory cells into the kidneys [40, 41].

During disease, kidneys are infiltrated by neutrophils and subsequently by monocytes, which differentiate into macrophages and contribute to tubular injury [43]. Proinflammatory macrophages are known to contribute to the initiation and progression of renal diseases [44–49], renal injury related to cisplatin nephrotoxicity [50, 51], and renal allograft injury [52, 53].

The main feature of renal fibrosis is an excessive production and accumulation of ECM (extracellular matrix) proteins, which leads to the formation of scar tissue and subsequently to renal dysfunction and organ failure [41, 54]. The implication is that M1 macrophages are responsible for triggering the fibrotic process due to their release of proinflammatory cytokines, which indirectly promote the proliferation of myofibroblasts and the recruitment of fibrocytes [21, 55].

TNF*α* is known to have an autocrine effect on the activation of macrophages [56] in a process which mediates kidney injury [57]. M1 macrophages release inflammatory mediators including ROS and TNF*α*, which augment an injury in a positive feedback loop, to cause renal fibrosis [1, 21]. Studies have shown that M1 proinflammatory macrophages are recruited into the kidney within the first hours after ischemia-reperfusion-induced acute kidney injury [58–60], whereas M2 anti-inflammatory macrophages predominate at a later time.

In the hypertensive animal model studied in this work, however, we found no increase in protein levels of TNF*α*, IL-1*β*, IL-6, IFN-*γ*, MCP-1, and iNOS in the kidneys. Thus, our data do not support the polarization of macrophages to an M1 phenotype, suggesting that at this stage of hypertension, there is no renal inflammatory process. We also found no increase in the expression of AT1R. Prior work in a model of rats that develop hypertension has shown that the infusion of Ang II increased the number of type 1 T helper (Th1) cytokine IFN-*γ*-secreting cells and decreased type 2 T helper (Th2) cytokine IL-4-secreting cells [61].

After the inflammatory phase orchestrated by the M1 phenotype, Th2 cytokines are produced and promote polarization to the M2 phenotype, which is known to create an anti-inflammatory environment [22, 43]. This response is generally associated with the resolution of inflammation and tissue healing. But when a lesion persists, these cells assume prorepair functions and promote irreversible fibrosis and the progressive destruction of renal tissue [21, 22].

M2 macrophages are initially anti-inflammatory, although the healing process depends on the termination of the initial injury [62]. In chronic conditions, on the other hand, M2 can activate resident fibroblasts through the release of transforming growth factor-beta (TGF-*β*), platelet-derived growth factor (PDGF), vascular endothelial growth factor (VEGF), insulin-like growth factor 1 (IGF-1), and galectin-3 [63, 64]. This suggests that the severity of fibrosis depends on the type of polarization macrophages undergo and the persistence of the inflammatory injury [21].

An elegant study by Ma et al. [65] suggested that after blocking AT1R with losartan, Ang II polarized macrophages into the M2 phenotype with a high expression of YM1/Chi3l3 and suppressed the expression of M1 markers in WT animals. It is important to note that this occurred in an obesogenic environment where the lean WT and AT1aKO animals showed no change in YM1/Chi3l3 protein levels.

Here, in contrast, we showed for the first time that the lean hypertensive animals presented macrophage polarization to the M2 phenotype with high levels of YM1/Chi3l3. Our transgenic animals come from a hypertensive environment and exhibit an overexpression of AOGEN.

In inflammation, the RAAS appears to act in an antagonistic way involving two different situations regarding the polarization of macrophages [9, 65], but the mechanisms have yet to be clarified. The activation of RAAS by AT1R in macrophages promotes the infiltration and activation of macrophages polarized to the M1 phenotype [1, 9, 65]. A systemic infusion of Ang II is known to induce the expression of proinflammatory mediators, such as MCP-1, TNF*α*, and IL-6, in vascular smooth muscle and kidney cells [66]. The other situation is related to M2 macrophages induced by Ang II stimulation. Moore et al. [32] confirmed Ang II-induced aortic infiltration with Ly6C^hi^ monocytes, but at 7-14 days, these cells began to express the M2 phenotype, with increased CD206 and arginase. In addition, macrophage-specific AT1R receptor deficiency exacerbates renal fibrosis induced by a unilateral ureteral obstruction [67].

**Figure 12:**
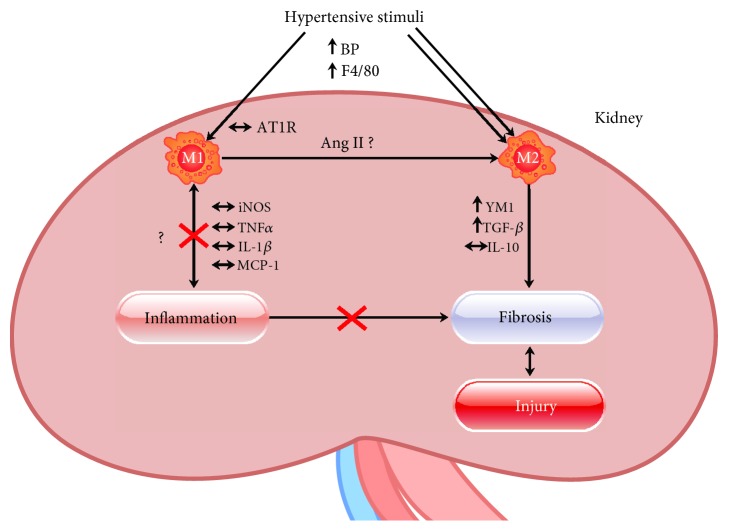
Macrophage polarization in the kidneys of 10- to 12-week-old hypertensive mice. The hypertensive stimuli from RAAS, as high levels of AOGEN contributed to macrophage polarization in the kidneys of hypertensive mice with a clear predominance of the M2 phenotype. The high levels of TGF-*β*1 may be involved in the development of fibrosis. Basal levels of IL-10 do not represent a form of protection against renal fibrosis in the hypertensive animals. In addition, at this stage of hypertension, our data do not support the polarization of macrophages to an M1 phenotype. It seems that the AT1aR in the kidneys from the hypertensive animals were not activated. High levels of AOGEN plus the hypertensive environment contribute to the M2 macrophage polarization. M2 macrophages produced high levels of YM1/Chi3l3 protein (91,89%).

In light of the data from our experiments, we suggest that at this stage of hypertension, elevated AOGEN levels contribute to the development of renal damage toward the predominance of the M2 phenotype. In our experiments, we did not block AT1aR, but the animals presented elevated levels of AOGEN, and it seems that the AT1aR in the kidneys from the hypertensive animals were not activated. This fact suggests that the hypertensive environment plus the increase of AOGEN contributes to M2 macrophage polarization. However, we cannot state that Ang II is the mediator of M2 macrophage polarization (Figures 3 and 12) in our animal model, since other members of the RAAS may be involved.

Some M2 markers, such as YM1/Chi3l3 from mice, were first identified as proteins that were secreted during infections by parasites and allergic inflammations [29, 68, 69]. It is known that YM1/Chi3l3 is a marker specific for the M2 macrophage phenotype [65], but little is known about its function in arterial hypertension and hypertensive nephropathy.

In this analysis, it has recently been shown that YKL-40, a member of the chitinase protein family, found in humans and homologous to YM1/Chi3l3, is positively associated with the incidence of hypertension among prehypertensive patients. The case-control study by Xu et al. [31] included an extraction of plasma samples from 20343 prehypertensive or normotensive Chinese subjects. This study suggested that YKL-40 may be a new biomarker for predicting hypertension in the prehypertensive population.

The analysis of the kidneys of our 10- to 12-week-old hypertensive mice revealed a chronic activation of macrophages with an M2 phenotype. In addition, we found significant increases in levels of YM1/Chi3l3 protein (91,89%) and collagen depositions.

Previous experiments by our group verified that the YM1/Chi3l3 gene was expressed in the hearts of these animals (unpublished data) but did not find significant differences in the hypertensive group compared to the control group at this stage. This suggests that at this point in the development of arterial hypertension, this protein is found in the kidney, but not in the heart of these animals, and may serve as a marker specific for hypertensive nephropathy.

Our data support the idea that M2 macrophages help promote the development of kidney fibrosis at a specific stage of hypertension; this is in agreement with studies [70, 71] pointing to macrophages as sources of profibrotic factors. TGF-*β*1 has already been identified as a central mediator of renal fibrosis [72–74] and plays an important role in the progression of CKD.

In contrast, studies of experimental kidney disease models have produced a body of evidence indicating a multifunctional role of TGF-*β* in inducing both profibrotic and protective effects [54]. This study did not reveal any protective effects from TGF-*β*1. On the contrary, our work suggests that high levels of TGF-*β*1 may be involved in the development of fibrosis.

This work also revealed that the kidneys of hypertensive animals experienced no change in IL-10. This cytokine is produced by several cell types, including macrophages, which polarize to an M2 phenotype, modulate the inflammatory response, and promote tissue repair [60, 75]. IL-10 is also known as an antifibrotic cytokine that is downregulated in CKD [76].

IL-10 controls inflammatory processes by suppressing the production of proinflammatory cytokines such as IL-1*β* and TNF*α*, which are known to be regulated by NF-*κ*B transcription [77–79]. In general, IL-10 improves vascular and renal functions in hypertension [80], although little has been reported on the effects of this cytokine on hypertension, particularly in immune environments that favor the development of fibrosis. Our work suggests that basal levels of IL-10 do not represent a form of protection against renal fibrosis in the hypertensive animals (Figure 12).

In conclusion, our work shows for the first time that hypertensive animals are predisposed to a polarization of macrophages to an M2 phenotype *in vitro*, revealing features that suggest a profibrotic profile. This fits with our findings that the kidneys of these hypertensive animals showed a high deposition of collagen, accompanied by an increase in the expression of macrophage markers with a clear predominance toward the M2 phenotype. Taken together, these data suggest that M2 macrophages, associated with high levels of YM1/Chi3l3, are linked to renal damage and fibrosis. Furthermore, it suggests that YM1/Chi3l3 may serve as a new biomarker of hypertensive nephropathy.

Future studies are needed involving both YM1/Chi3l3 in mice and YKL-40 in humans at different time points to confirm whether reducing levels of these proteins may be beneficial in delaying the development of hypertensive nephropathy.

## Figures and Tables

**Table 1:** The gene-specific primer sequences.

Gene name	Direction	Primer sequence (5′-3′)
*β*-Actin	Forward	CTG GCC TCA CTG TCC ACC TT
	Reverse	CGG ACT CAT CGT ACT CCT GCT T

iNOS	Forward	CTG CTG GTG GTG ACA AGC ACA TTT
	Reverse	ATG TCA TGA GCA AAG GCG CAG AAC

F4/80	Forward	CTTTGGCTATGGGCTTCCAGTC
	Reverse	GCAAGGAGGACAGAGTTTATCGTG

CD86	Forward	TCT CCA CGG AAA CAG CAT CT
	Reverse	CTT ACG GAA GCA CCC ATG AT

TNF*α*	Forward	CCC ACG TCG TAG CAA ACC AC
	Reverse	CAC AGA GCA ATG ACT CCA AAG TAG

IL-1*β*	Forward	GGC TCA TCT GGG ATC CTC TC
	Reverse	TCA TCT TTT GGG GTC CGT CA

MMP-9	Forward	ACG GAC CCG AAG CGG ACA TT
	Reverse	TTG CCC AGC GAC CAC AAC TC

IL-6	Forward	TAGTCCTTCCTACCCCAATTTCC
	Reverse	TTGGTCCTTAGCCACTCCTCC

CD206	Forward	CAA GGA AGG TTG GCA TTT GT
	Reverse	CCT TTC AGT CCT TTG CAA GC

Collagen I	Forward	GAC ATG TTC AGC TTT GTG GAC CTC
	Reverse	GGG ACC CTT AGG CCA TTG TGT A

Fibronectin	Forward	CCT ACG GCC ACT GTG TCA CC
	Reverse	AGT CTG GGT CAC GGC TGT CT

KIM-1	Forward	TGT CGA GTG GAG ATT CCT GGA TGG T
	Reverse	GGT CTT CCT GTA GCT GTG GGC C

YM1	Forward	CCC CTG GAC ATG GAT GAC TT
	Reverse	AGC TCC TCT CAA TAA GGG CC

TGF-*β*1	Forward	CAA CAA TTC CTG GCG TTA CCT TGG
	Reverse	GAA AGC CCT GTA TTC CGT CTC CTT

MCP-1	Forward	CTCACCTGCTGCTACTCATTC
	Reverse	TTACGGCTCAACTTCACATTCA

Collagen III	Forward	TCCTAACCAAGGCTGCAAGATGGA
	Reverse	AGGCCAGCTGTACATCAAGGACAT

AT1a	Forward	CAAGTCGCACTCAAGCCTG
	Reverse	CTCAGAACAAGACGCAGGC

## Data Availability

We will send the information if necessary. All authors Paula Andréa Malveira Cavalcante, Natalia Alenina, Alexandre Budu, Leandro Ceotto Freitas-Lima, Thaís Alves-Silva, Juan Sebastian Henao Agudelo, Fatimunnisa Qadri, Niels Olsen Saraiva Camara, Michael Bader, and Ronaldo Carvalho Araújo declare that all data as real-time quantitative PCR, histology, enzyme-linked immunosorbent assay (ELISA), Western blot, cytokine assessment in kidney sample (CBA), and immunofluorescence used to support the findings of this study are included within the article.
